# Supplementary material for: A Likelihood Approach for Real-Time Calibration of Stochastic Compartmental Epidemic Models
Source: PLoS Comput Biol. 2017 Jan 17;13(1):e1005257. doi: 10.1371/journal.pcbi.1005257 (PMC5240920; doi:10.1371/journal.pcbi.1005257)
Supplement: S1 File — (TAR.GZ) [file pcbi.1005257.s014.tar.gz › HSPH_Online-SI-Revision/MSS10/n10-severe/n10-severe_NEW_table0.pdf]

1	R0	{1.68952, 11.8402, 1.}	{1.7434, 11.5667, 2.}	{{1.7434, 11.5667, 2.}, {1.7434, 11.5667, 2.}}	{3.18914, 2.30994, 100.}	inf	1046.	1120.43	{1082.8, 1154.01}	7.11557	0.029697	newly	589.	654.39	{622., 684.}	11.1154	0.0454585	Reff	0.898487	0.914165	{0.906696, 0.920583}	1.74494	ar	6872.	7141.55	{7017., 7270.}	3.92244
2	R0	{1.71739, 9.62661, 0.1}	{1.44742, 4.13437, 0.27}	{{1.44762, 4.00571, 0.}, {1.44762, 4.94504, 2.}}	{15.7196, 57.0527, 348.}	inf	945.	407.641	{334.942, 471.071}	56.8634	0.368663	newly	711.	622.36	{524., 720.}	13.6118	0.0654837	Reff	0.984408	0.90744	{0.897938, 0.918089}	7.81869	ar	6874.	5228.07	{4978., 5478.}	23.9443
3	R0	{1.48936, 6.23975, 0.1}	{1.86752, 12.6342, 3.7}	{{1.454, 5.4396, 3.}, {1.94617, 14.0718, 4.}}	{26.1367, 108.392, 3608.}	inf	625.	1190.82	{511.677, 1361.6}	98.4377	0.295728	newly	640.	646.35	{602., 684.}	4.69844	0.0205904	Reff	0.888701	1.00301	{0.89651, 1.03126}	13.0333	ar	5666.	7351.15	{5487., 7830.}	31.1696
4	R0	{1.62394, 7.51135, 1.}	{1.48315, 5.3188, 1.9}	{{1.44762, 4.00571, 0.}, {1.58886, 7.29751, 3.}}	{8.66956, 29.1898, 142.}	inf	753.	528.47	{363.015, 732.171}	31.0375	0.171902	newly	670.	630.74	{548., 706.}	8.8806	0.0411463	Reff	0.945133	0.895955	{0.883258, 0.917633}	5.20327	ar	6417.	5622.83	{5127., 6257.}	12.4187
5	R0	{1.53632, 8.57652, 1.}	{1.44228, 5.95856, 0.09}	{{1.44485, 6.00596, 0.}, {1.44485, 6.00596, 0.}}	{6.12085, 30.5247, 97.}	inf	707.	474.323	{423.367, 515.19}	32.9105	0.174662	newly	572.	520.93	{476., 562.}	9.07168	0.0421506	Reff	0.929627	0.906268	{0.900631, 0.912703}	2.51273	ar	6260.	5366.77	{5183., 5559.}	14.2688
6	R0	{1.47166, 5.7434, 0.1}	{1.33937, 4.09188, 0.}	{{1.24751, 2.5364, 0.}, {1.78225, 11.7348, 0.}}	{16.0222, 63.4242, 100.}	inf	531.	376.646	{153.755, 1137.16}	67.8166	0.388854	newly	615.	516.87	{336., 700.}	23.478	0.124099	Reff	0.960847	0.888294	{0.84325, 1.05168}	10.7772	ar	5650.	4557.13	{3659., 7173.}	28.9807
7	R0	{1.48179, 8.87369, 0.1}	{1.38622, 6.64078, 0.02}	{{1.38815, 6.71734, 0.}, {1.38815, 6.71734, 0.}}	{6.44964, 25.1633, 116.}	inf	480.	359.226	{324.934, 401.692}	25.1613	0.130636	newly	360.	346.89	{308., 389.}	8.28056	0.0376865	Reff	0.902706	0.861184	{0.85532, 0.866221}	4.59982	ar	5233.	5005.81	{4837., 5191.}	4.44582
8	R0	{1.53669, 6.47807, 0.1}	{1.93427, 12.3169, 2.06}	{{1.62191, 8.12461, 2.}, {2.01902, 13.4708, 2.}}	{26.3226, 91.6011, 1960.}	inf	675.	1353.61	{813.046, 1540.98}	102.697	0.297501	newly	686.	747.05	{666., 797.}	10.5	0.0433092	Reff	0.902344	0.99936	{0.924847, 1.02751}	10.9644	ar	5934.	7632.28	{6441., 8051.}	29.2208
9	R0	{1.57227, 6.20989, 0.1}	{1.26191, 2.63423, 0.06}	{{1.24751, 2.5364, 0.}, {1.24751, 2.5364, 0.}}	{19.7397, 57.5801, 156.}	inf	741.	282.934	{175.767, 386.818}	61.8173	0.436208	newly	787.	630.77	{438., 800.}	21.6734	0.115037	Reff	1.05279	0.902523	{0.884505, 0.908184}	14.2734	ar	6136.	4105.08	{3650., 4409.}	33.0984
10	R0	{1.54936, 6.50938, 0.1}	{1.45037, 4.00724, 0.}	{{1.44762, 4.00571, 0.}, {1.44762, 4.00571, 0.}}	{6.38907, 38.4391, 100.}	inf	717.	422.454	{350.078, 506.48}	41.0803	0.234018	newly	740.	641.39	{518., 755.}	14.9986	0.0734617	Reff	0.879107	0.865666	{0.85048, 0.880901}	1.68393	ar	6275.	5345.19	{5023., 5589.}	14.8177
11	R0	{1.42179, 3.18601, 0.1}	{1.39577, 2.12385, 0.47}	{{1.37363, 1.89883, 0.}, {1.43965, 2.81014, 3.}}	{2.25804, 33.7477, 520.}	inf	449.	256.635	{106.181, 375.161}	43.4672	0.295361	newly	893.	706.72	{323., 1006.}	27.4513	0.167272	Reff	0.921746	0.931689	{0.905472, 0.955953}	1.94186	ar	5322.	4575.88	{3881., 5066.}	14.062
12	R0	{1.48035, 6.46183, 0.1}	{1.47828, 4.51291, 0.21}	{{1.44762, 4.00571, 0.}, {1.44762, 4.00571, 0.}}	{4.9806, 42.3189, 296.}	inf	718.	509.66	{347.028, 551.94}	42.6557	0.223704	newly	750.	761.09	{607., 871.}	10.66	0.0474685	Reff	0.99065	1.01639	{0.996662, 1.02462}	3.37321	ar	5793.	5424.57	{4926., 5665.}	11.5638
13	R0	{1.75755, 8.53854, 1.}	{1.64223, 6.44363, 0.4}	{{1.44762, 4.00571, 0.}, {1.68297, 7.5059, 1.}}	{15.7792, 44.3658, 70.}	inf	1074.	846.586	{409.812, 1063.37}	48.175	0.252843	newly	871.	816.82	{648., 959.}	11.3708	0.0535834	Reff	0.980361	0.933907	{0.888061, 0.969008}	6.36135	ar	6832.	6264.34	{5240., 7071.}	15.195
14	R0	{1.47355, 4.89451, 0.1}	{1.45502, 4.01531, 0.}	{{1.44762, 4.00571, 0.}, {1.47488, 4.06661, 0.}}	{1.3114, 17.963, 100.}	inf	528.	456.544	{380.06, 527.445}	14.3487	0.0699086	newly	648.	691.68	{580., 789.}	11.5679	0.0478434	Reff	0.862321	0.861855	{0.850516, 0.874382}	0.882012	ar	5710.	5476.08	{5265., 5723.}	4.39405
15	R0	{1.62071, 5.98665, 0.1}	{1.48181, 3.94572, 0.03}	{{1.47488, 3.83841, 0.}, {1.53501, 4.06661, 0.}}	{8.57033, 34.0913, 128.}	inf	906.	548.273	{466.286, 614.503}	39.4842	0.221341	newly	967.	841.55	{727., 953.}	13.5915	0.0655937	Reff	0.893659	0.870069	{0.856242, 0.895426}	2.85947	ar	6635.	5710.46	{5468., 5946.}	13.9343
16	R0	{1.67268, 7.7256, 1.}	{2.58526, 19.0366, 3.91}	{{2.64373, 19.7855, 4.}, {2.64373, 19.7855, 4.}}	{54.5582, 146.806, 291.}	inf	927.	2562.99	{2631.69, 2713.81}	177.847	0.436745	newly	767.	882.22	{847., 925.}	16.2634	0.0655814	Reff	0.766087	0.751458	{0.738174, 0.760154}	2.58037	ar	6947.	9038.1	{9101., 9223.}	30.3207
17	R0	{1.67805, 11.1417, 1.}	{1.3831, 5.02408, 0.11}	{{1.24751, 2.5364, 0.}, {1.44485, 6.00596, 0.}}	{17.5769, 54.9074, 95.}	inf	929.	425.1	{169.663, 569.212}	54.2412	0.380464	newly	581.	544.18	{396., 635.}	12.5577	0.0675134	Reff	1.07479	0.954373	{0.886263, 0.991185}	11.2038	ar	6625.	4912.2	{3643., 5578.}	25.8536
18	R0	{1.66743, 9.99046, 0.1}	{1.44485, 6.00596, 0.}	{{1.44485, 6.00596, 0.}, {1.44485, 6.00596, 0.}}	{13.3488, 39.883, 100.}	inf	904.	500.03	{464.565, 537.406}	44.6869	0.258043	newly	639.	550.95	{505., 597.}	13.7793	0.0654437	Reff	0.998457	0.923542	{0.918112, 0.928612}	7.50308	ar	6899.	5394.05	{5232., 5578.}	21.814
19	R0	{1.70787, 9.85478, 0.1}	{1.45499, 4.01987, 0.}	{{1.44762, 4.00571, 0.}, {1.47488, 4.06661, 0.}}	{14.8066, 59.2089, 100.}	inf	940.	368.673	{313.89, 424.614}	60.7794	0.409365	newly	617.	544.03	{461., 618.}	13.0551	0.0624749	Reff	0.877333	0.830552	{0.81972, 0.843772}	5.33219	ar	6867.	5368.33	{5153., 5560.}	21.8242
20	R0	{1.53809, 10.5848, 1.}	{1.30806, 4.57129, 0.08}	{{1.24751, 2.5364, 0.}, {1.38815, 6.71734, 0.}}	{14.9554, 56.8127, 100.}	inf	660.	291.835	{155.756, 449.48}	55.7826	0.392415	newly	432.	415.4	{306., 497.}	13.0926	0.0639828	Reff	1.05928	0.948374	{0.913019, 0.991031}	10.4702	ar	6108.	4238.58	{3498., 4999.}	30.6061
21	R0	{1.59925, 6.74479, 0.1}	{1.45336, 4.01622, 0.}	{{1.44762, 4.00571, 0.}, {1.47488, 4.06661, 0.}}	{9.12259, 40.4545, 100.}	inf	837.	455.223	{388.962, 516.467}	45.6125	0.267028	newly	789.	702.56	{609., 800.}	12.0279	0.0571567	Reff	0.912212	0.884474	{0.873089, 0.894922}	3.04076	ar	6411.	5396.87	{5176., 5591.}	15.8186
22	R0	{1.38779, 4.40977, 0.1}	{1.78242, 12.5539, 3.97}	{{1.24751, 2.5364, 0.}, {1.92096, 15.109, 5.}}	{32.2652, 199.18, 3908.}	inf	333.	1051.11	{187.326, 1307.58}	230.659	0.50688	newly	507.	578.	{482., 630.}	20.4063	0.0849647	Reff	0.993103	1.13593	{0.911467, 1.20063}	17.6075	ar	5154.	6910.23	{3905., 7818.}	44.2835
23	R0	{1.53657, 7.50352, 1.}	{1.36782, 5.09407, 1.}	{{1.3618, 4.98002, 1.}, {1.40191, 5.7404, 1.}}	{10.9823, 32.1109, 0.}	inf	535.	366.728	{305.042, 429.474}	31.4527	0.167735	newly	479.	464.53	{399., 532.}	9.34656	0.0421332	Reff	0.991395	0.905495	{0.896287, 0.916108}	8.66451	ar	5928.	4818.5	{4562., 5103.}	18.7163
24	R0	{1.71131, 6.24526, 0.1}	{1.51474, 4.0309, 0.07}	{{1.47488, 3.83841, 0.}, {1.53501, 4.06661, 0.}}	{11.4863, 35.4567, 164.}	inf	964.	620.33	{535.99, 713.762}	35.8358	0.195614	newly	1016.	968.29	{854., 1095.}	8.64469	0.0392838	Reff	0.982634	0.92165	{0.896325, 0.944863}	6.23998	ar	6924.	5879.12	{5573., 6171.}	15.0907
25	R0	{1.4412, 3.07729, 0.1}	{1.4287, 2.44137, 0.69}	{{1.39162, 1.89883, 0.}, {1.43965, 2.60601, 1.}}	{1.52972, 22.6814, 662.}	inf	606.	442.974	{261.864, 597.55}	28.8846	0.168142	newly	1165.	1089.74	{807., 1360.}	16.0876	0.0822186	Reff	0.935195	0.95009	{0.929656, 0.967155}	1.89277	ar	5810.	5159.03	{4663., 5603.}	11.7127
26	R0	{1.4159, 4.93858, 0.1}	{1.2498, 2.72216, 0.03}	{{1.24751, 2.5364, 0.}, {1.25474, 3.26445, 0.}}	{11.731, 44.8796, 128.}	inf	458.	223.223	{152.025, 298.721}	51.2614	0.32737	newly	608.	481.25	{345., 631.}	23.8076	0.125708	Reff	0.962104	0.878577	{0.868027, 0.886372}	8.6817	ar	5255.	3942.67	{3613., 4283.}	24.973
27	R0	{1.57465, 7.05414, 0.1}	{1.3492, 3.6437, 0.44}	{{1.24751, 2.5364, 0.}, {2.12117, 12.1677, 4.}}	{22.4792, 65.8319, 518.}	inf	797.	443.31	{212.83, 1591.24}	69.4753	0.441152	newly	776.	697.73	{501., 949.}	19.3286	0.0949051	Reff	1.05486	0.938649	{0.890374, 1.22419}	15.0317	ar	6359.	4505.34	{3703., 8149.}	36.0245
28	R0	{1.41422, 4.62402, 0.1}	{2.97157, 19.3547, 2.}	{{2.97157, 19.3547, 2.}, {2.97157, 19.3547, 2.}}	{110.121, 318.569, 1900.}	inf	570.	2889.25	{2832.46, 2941.03}	406.886	0.704859	newly	804.	1080.49	{1040., 1116.}	34.3893	0.128182	Reff	0.944699	1.29583	{1.28043, 1.31216}	37.1683	ar	5485.	9395.83	{9348., 9440.}	71.3005
29	R0	{1.41901, 3.63017, 0.1}	{1.52472, 3.97812, 0.}	{{1.47608, 3.83841, 0.}, {1.53501, 4.02511, 0.}}	{7.48842, 10.5388, 100.}	inf	600.	646.838	{549.067, 731.629}	11.5226	0.0475147	newly	1020.	1010.06	{884., 1125.}	7.09412	0.0313965	Reff	0.854528	0.910986	{0.888173, 0.928971}	6.60695	ar	5979.	5965.26	{5718., 6162.}	2.55461
30	R0	{1.60719, 4.72984, 0.1}	{1.71958, 5.43705, 0.03}	{{1.53501, 4.02511, 0.}, {1.74592, 5.83416, 0.}}	{7.98093, 18.23, 124.}	inf	850.	1018.93	{828.21, 1127.76}	23.1485	0.0908677	newly	1189.	1266.36	{1163., 1369.}	8.0656	0.0334084	Reff	0.978457	1.01715	{0.965532, 1.03887}	4.50594	ar	6479.	6941.95	{6205., 7192.}	8.62896
31	R0	{1.38175, 6.26468, 0.1}	{1.24751, 2.5364, 0.}	{{1.24751, 2.5364, 0.}, {1.24751, 2.5364, 0.}}	{9.715, 59.5126, 100.}	inf	497.	205.829	{141.825, 260.54}	58.5856	0.397593	newly	506.	442.56	{303., 553.}	18.8103	0.0962422	Reff	0.889156	0.839099	{0.831883, 0.846914}	5.62979	ar	5397.	4024.63	{3767., 4312.}	25.4284
32	R0	{1.4947, 8.8847, 1.}	{1.35153, 5.29515, 1.12}	{{1.28596, 3.98513, 0.}, {1.36632, 6.77341, 2.}}	{9.57883, 40.4014, 50.}	inf	437.	280.703	{217.35, 359.035}	35.7659	0.201228	newly	341.	337.01	{286., 383.}	8.98827	0.039898	Reff	0.948387	0.878555	{0.849203, 0.892171}	7.36328	ar	5407.	4588.7	{4269., 4923.}	15.1341
33	R0	{1.55891, 8.28978, 1.}	{1.41782, 6.1715, 1.}	{{1.3618, 4.98002, 1.}, {1.51295, 8.4085, 1.}}	{9.05045, 27.6236, 0.}	inf	655.	439.727	{320.096, 661.434}	33.8081	0.192045	newly	521.	459.28	{402., 519.}	12.5451	0.0600389	Reff	0.96668	0.909021	{0.887303, 0.941764}	5.96469	ar	6239.	5182.82	{4722., 5984.}	16.9835
34	R0	{1.60764, 10.4729, 0.1}	{1.51295, 8.4085, 1.}	{{1.51295, 8.4085, 1.}, {1.51295, 8.4085, 1.}}	{5.89003, 19.7119, 900.}	inf	772.	610.836	{565.984, 653.416}	20.8761	0.102296	newly	469.	471.47	{430., 509.}	4.6887	0.0203364	Reff	0.879861	0.85242	{0.845908, 0.85879}	3.11877	ar	6495.	5959.6	{5808., 6126.}	8.24326
35	R0	{1.48527, 5.74485, 1.}	{2.46976, 17.5057, 3.}	{{2.46976, 17.5057, 3.}, {2.46976, 17.5057, 3.}}	{66.2834, 204.719, 200.}	inf	650.	2237.98	{2192.03, 2283.14}	244.305	0.536879	newly	769.	913.11	{879., 947.}	18.7399	0.0744037	Reff	0.956217	1.19784	{1.18642, 1.209}	25.2683	ar	5811.	8887.6	{8817., 8950.}	52.9444
36	R0	{1.61457, 9.93364, 1.}	{1.48344, 7.30408, 1.55}	{{1.3618, 4.98002, 1.}, {1.56097, 8.77207, 2.}}	{8.12183, 26.4713, 59.}	inf	799.	597.773	{377.192, 768.161}	25.263	0.145941	newly	557.	547.51	{470., 616.}	8.25673	0.0372204	Reff	1.02993	0.974632	{0.92269, 1.01077}	5.3695	ar	6338.	5635.28	{4729., 6304.}	11.1524
37	R0	{1.47831, 7.08139, 0.1}	{1.24736, 2.54904, 0.}	{{1.24751, 2.5364, 0.}, {1.24751, 2.5364, 0.}}	{15.6223, 64.0037, 100.}	inf	564.	193.819	{105.251, 264.576}	65.635	0.489318	newly	542.	436.34	{221., 603.}	24.8967	0.142413	Reff	0.993129	0.884156	{0.874867, 0.895309}	10.9726	ar	5530.	3791.78	{3336., 4130.}	31.4325
38	R0	{1.73445, 9.8858, 0.1}	{1.87692, 11.8599, 3.9}	{{1.85898, 11.7144, 3.}, {1.85898, 11.7144, 4.}}	{8.21389, 19.9689, 3800.}	inf	986.	1310.24	{1241.75, 1371.56}	32.884	0.122832	newly	672.	769.76	{731., 808.}	14.5476	0.0586639	Reff	1.0313	1.05482	{1.03995, 1.06538}	2.28067	ar	6842.	7582.84	{7434., 7734.}	10.8278
39	R0	{1.66621, 11.4824, 0.1}	{1.4536, 6.99067, 1.}	{{1.40191, 5.7404, 1.}, {1.51295, 8.4085, 1.}}	{12.7599, 39.1184, 900.}	inf	863.	523.682	{369.094, 692.678}	39.3184	0.232065	newly	517.	493.48	{421., 564.}	9.22631	0.0424829	Reff	1.00256	0.923209	{0.90112, 0.945064}	7.91471	ar	6473.	5432.74	{4869., 6024.}	16.0708
40	R0	{1.60694, 9.95741, 1.}	{1.49228, 6.7887, 0.45}	{{1.41571, 4.98002, 0.}, {1.78373, 11.799, 2.}}	{10.8761, 38.111, 99.}	inf	852.	583.967	{404.301, 1120.5}	42.4294	0.238338	newly	560.	551.14	{478., 642.}	8.90357	0.039125	Reff	0.960147	0.92818	{0.905538, 1.01486}	5.33363	ar	6625.	5643.95	{5091., 7233.}	17.8854
41	R0	{1.55128, 4.71911, 0.1}	{1.47664, 3.98786, 0.}	{{1.47488, 3.83841, 0.}, {1.47608, 4.06661, 0.}}	{4.81177, 15.4956, 100.}	inf	710.	588.788	{492.067, 669.293}	17.5524	0.0863862	newly	1008.	976.85	{848., 1119.}	8.79861	0.0397084	Reff	1.00632	0.975789	{0.957302, 0.991918}	3.09447	ar	6141.	5685.4	{5450., 5966.}	7.41899
42	R0	{1.60063, 6.81118, 0.1}	{1.45223, 4.00581, 0.03}	{{1.44762, 4.00571, 0.}, {1.47488, 4.00571, 0.}}	{9.27123, 41.1878, 128.}	inf	843.	462.678	{398.588, 531.756}	45.1153	0.263252	newly	835.	718.73	{633., 823.}	14.2695	0.0689619	Reff	0.933487	0.902163	{0.889857, 0.913345}	3.3556	ar	6528.	5367.97	{5113., 5562.}	17.7701
43	R0	{1.56986, 5.44652, 0.1}	{1.46929, 3.9854, 0.}	{{1.44762, 3.83841, 0.}, {1.47608, 4.06661, 0.}}	{6.40644, 26.8267, 100.}	inf	706.	514.078	{443.157, 583.942}	27.1844	0.140277	newly	846.	788.34	{671., 898.}	9.88889	0.0460191	Reff	0.905652	0.875821	{0.863229, 0.88944}	3.29392	ar	6312.	5588.51	{5359., 5816.}	11.4621
44	R0	{1.47523, 8.07785, 0.1}	{1.39108, 5.5351, 1.}	{{1.3618, 4.98002, 1.}, {1.40191, 5.7404, 1.}}	{5.70421, 31.4781, 900.}	inf	601.	412.491	{344.38, 473.57}	31.3658	0.166496	newly	507.	490.48	{433., 543.}	7.49112	0.0337744	Reff	0.965243	0.936354	{0.920512, 0.946652}	2.99289	ar	5692.	4968.57	{4692., 5219.}	12.7096
45	R0	{1.6417, 7.69429, 0.1}	{1.59879, 6.13076, 0.87}	{{1.44762, 4.00571, 0.}, {1.68297, 7.5059, 2.}}	{6.67848, 23.5175, 852.}	inf	911.	761.028	{415.142, 991.613}	25.7851	0.144243	newly	777.	778.58	{629., 887.}	10.4813	0.0470323	Reff	0.893577	0.889055	{0.849379, 0.915914}	2.67661	ar	6735.	6289.95	{5342., 6941.}	9.20817
46	R0	{1.38865, 5.83473, 0.1}	{1.27979, 3.81324, 0.07}	{{1.25474, 3.26445, 0.}, {1.28596, 3.98513, 0.}}	{7.83923, 34.6458, 162.}	inf	383.	252.824	{186.056, 315.106}	34.2196	0.191209	newly	442.	423.72	{333., 511.}	14.3213	0.0655086	Reff	0.981081	0.920787	{0.905604, 0.931337}	6.14571	ar	5233.	4076.91	{3672., 4380.}	22.0923
47	R0	{1.45289, 6.87903, 0.1}	{1.31194, 4.28139, 0.44}	{{1.28596, 3.98513, 0.}, {1.3618, 4.98002, 1.}}	{9.7014, 37.7617, 460.}	inf	477.	311.598	{243.277, 381.057}	34.6755	0.192754	newly	487.	473.6	{382., 559.}	11.1253	0.0504961	Reff	1.01252	0.935839	{0.909091, 0.967253}	7.57321	ar	5379.	4367.69	{3986., 4834.}	18.8011
48	R0	{1.69416, 9.4831, 0.1}	{1.74819, 9.55212, 0.3}	{{1.58886, 7.29751, 0.}, {1.81647, 10.5184, 1.}}	{6.91834, 14.5561, 340.}	inf	944.	1113.38	{771.933, 1292.18}	27.616	0.108522	newly	702.	806.25	{721., 878.}	15.349	0.0611827	Reff	1.03412	1.03529	{0.990336, 1.06047}	2.4498	ar	6925.	7107.25	{6280., 7517.}	7.53718
49	R0	{1.47843, 7.16286, 0.1}	{1.24805, 2.54216, 0.03}	{{1.24751, 2.5364, 0.}, {1.24751, 2.5364, 0.}}	{15.5829, 64.5092, 128.}	inf	577.	215.42	{145.085, 295.007}	62.6655	0.444278	newly	548.	477.78	{343., 632.}	19.7701	0.10341	Reff	0.972659	0.866216	{0.855683, 0.874621}	10.9435	ar	5449.	3930.12	{3610., 4196.}	27.8745
50	R0	{1.68843, 5.72427, 0.1}	{1.53383, 4.02137, 0.}	{{1.53501, 4.02511, 0.}, {1.53501, 4.02511, 0.}}	{9.15643, 29.7487, 100.}	inf	853.	563.422	{479.967, 633.667}	33.9482	0.182348	newly	913.	819.02	{706., 903.}	11.218	0.0530781	Reff	0.853333	0.819604	{0.808126, 0.832077}	3.9526	ar	6800.	6024.56	{5824., 6224.}	11.4035
